# Metabolomics analysis reveals cytotoxic effects of ouabain towards psoriatic keratinocytes via impairment of glutathione metabolism

**DOI:** 10.1007/s00438-023-02001-9

**Published:** 2023-03-01

**Authors:** Xuan Zhou, Fei Fei, Wei Song, Hehua Ma, Zhenzhen Xu, Jing Yue, Bei Cao, Runbin Sun, Yu Zhao, Yuanxun Yang, Junyi Jiang, Yan Geng, Zuyi Weng, Juan Li

**Affiliations:** Phase I Clinical Trials Unit, The Affiliated Drum Tower Hospital of Nanjing University Medical School, 359 Pu Zhu Middle Road, Nanjing, 210031 China

**Keywords:** Keratinocyte, Ouabain, Metabolomics, Glutathione metabolism, Psoriasis

## Abstract

Ouabain is a cardiac glycoside long studied for treating heart diseases, but the attempts to evaluate its anti-psoriatic activity have not been reported. We aimed to explore the effects of ouabain on proliferation and metabolism towards psoriatic keratinocytes. In human HaCaT keratinocytes, ouabain potently decreased viability, promoted apoptosis and caused G2/M cycle arrest. Metabolomics analysis indicated that ouabain markedly impaired glutathione metabolism. The solute carrier family 7 member 11 (SLC7A11) is an amino acid transporter highly specific to cysteine, which is critical for glutathione synthesis. Ouabain downregulated SLC7A11, reduced cysteine uptake and subsequently inhibited glutathione synthesis, probably through inhibiting Akt/mTOR/beclin axis that regulate protein activity of SLC7A11. The impaired glutathione synthesis and oxidative stress caused by ouabain may contribute to its cytotoxicity towards psoriatic keratinocytes. Our results provide experimental evidence supporting further study of ouabain as a potential anti-psoriatic agent.

## Introduction

Psoriasis is a multifactorial skin disease characterized by epidermal hyperproliferation and chronic inflammation (Zampetti et al. 2009; Griffiths et al. 2021). Traditional treatments are effective by counteracting keratinocyte hyperproliferation, but unfavorable side effects limit their clinical use (Agrawal et al. 2013). Recently developed biologics effectively alleviate symptoms, but are at a high cost and none of them can offer a curative treatment for psoriasis (Jadon et al. 2020; Griffiths et al. 2021). Thus, it is still urgent to identify new therapeutic targets.

Ouabain, a plant-derived cardiac glycoside, has been most studied for cardiovascular diseases due to its positive cardiac inotropic effects by inhibiting Na + /K + − ATPase-mediated ion transport (Li et al. 2010). Recent evidence shows that low molar concentrations of ouabain also exhibit anti-proliferative and anti-inflammatory actions in several cancer cell lines (Du et al. 2021). Since epidermal hyperproliferation and chronic inflammation are the main characteristics of psoriasis, one would speculate that ouabain would also be therapeutically effective against psoriasis. However, the attempts to evaluate the anti-psoriatic activity of ouabain have never been reported.

Psoriasis is often associated with metabolic disorders, such as atherosclerosis and type II diabetes (Griffiths et al. 2021). Several studies have suggested that metabolic reprogramming may participate in the pathogenesis of psoriasis (Bandyopadhyay and Larregina 2020; Lian et al. 2020). Early reports usually measured levels of several single metabolic compounds using commercialized kits, such as lactate and glucose, but none of these studies provide a comprehensive view of the full metabolic pattern of psoriatic keratinocytes. The novel metabolomics research is the analysis of the full metabolome profile of an organism, yielding thousands of metabolites and providing comprehensive information of biological systems under a given set of conditions (Eckhart et al. 2012). Several studies have utilized metabolomics approach to examine metabolite differences in psoriasis, which have greatly increased our understanding of the physiological processes underlying this complex disease (Armstrong et al. 2014; Koussiouris et al. 2021).

In this study, we explored the effects and mechanisms of ouabain on cellular proliferation and metabolism using untargeted gas chromatography-mass spectrometry (GC/MS) metabolomics approach in human HaCaT keratinocytes, a rapidly proliferating cell line commonly used as an in vitro model for studying psoriasis (Zampetti et al. 2009). To the best of our knowledge, we report for the first time that ouabain significantly reduced proliferation and caused G2/M cell cycle arrest in keratinocytes. Metabolomics data indicated that ouabain markedly impaired cellular metabolism, in particular glutathione (GSH) metabolism. The solute carrier family 7 member 11 (SLC7A11) is an amino acid transporter highly specific to cysteine, which is critical for glutathione synthesis (Jyotsana et al. 2022). Ouabain significantly reduced intracellular cysteine availability by downregulating its transporter SLC7A11 and subsequently decreased GSH synthesis while increased oxidative stress, probably via inhibition of the Akt/mTOR/beclin axis. The impaired GSH metabolism and redox balance caused by ouabain may contribute to its cytotoxic activities toward psoriatic keratinocytes. Our study provides preliminary evidence supporting further study of ouabain as a potential anti-psoriatic agent.

## Materials and methods

### Human keratinocyte culture

The immortalized human keratinocyte cell line, HaCaT, was kindly provided by Dr. Qian Tan from Department of Burns and Plastic Surgery in Nanjing Drum Tower Hospital. HaCaT cells were cultured in DMEM supplemented with 10% FBS and 1% penicillin/streptomycin (Sigma-Aldrich, Wuxi, China). Cells were maintained at 37 °C in a humidified atmosphere with 5% CO_2_.

### Cell proliferation assay

HaCaT cells (1 × 10^5^ cells per well) were seeded in 6-well plates. At 24 h (h) after ouabain (O3125, Sigma-Aldrich, MO, USA) treatment at various concentrations, cell proliferation was measured using the cell counting kit-8 (CCK-8)-based assay kit (96,992, Sigma-Aldrich). Cell viability was determined by trypan-blue (0.4%) exclusion test.

### Cell apoptosis assay

HaCaT cells (1 × 10^5^ cells per well) were seeded in 6-well plates. At 24 h after ouabain treatment, the number of apoptotic cells was measured by flow cytometry using an assay kit (APOAF, Sigma-Aldrich) according to the instructions.

### Cell cycle analysis

HaCaT cells (1 × 10^5^ cells per well) were seeded in 6-well plates. After ouabain treatment (0–200 nM, 24 h), cells were trypsinized, collected, and washed with phosphate-buffered saline. The percentage of cells that were in G0/G1, S and G2/M phases were determined by flow cytometry using an assay kit (ab112116, Abcam, MA, USA) according to the instructions provided.

### Untargeted GC/MS metabolomics analysis

#### Cell treatment and harvest

HaCaT cells (1 × 10^6^ cells per well) were seeded in six-well plates. After ouabain treatment (100 nM, 24 h), culture supernatants were collected and the remaining cells were washed with ice-cold saline before addition of 400 μL purified water (Milli-Q system, Millipore, Bedford, USA). The culture media and the plates were then stored at − 80 °C until extraction.

#### Sample preparation and GC/MS analysis

For extraction of intracellular metabolites, the cell samples were harvested after three freeze–thaw cycles. Then 900 μL methanol containing (^13^C_2_)-myristic acid (1.5 μg/mL) as an internal standard (IS) was added, and the methanol-cell mixture was transferred to an eppendorf tube. For extraction of extracellular metabolites in culture media, 100μL of culture media were extracted with 300 μL methanol containing IS (0.5 μg/mL). After vortexing and centrifuging (20,000 g, 10 min, 4 °C), the supernatants from both the cell lysates and media were evaporated to dryness, and then oximated with 30 μL of pyridine containing 10 mg/mL methoxyamine for 16 h at room temperature. The mixture was then derivatized with 30 μL of MSTFA + 1% TMCS for 1 h. Then, 30 μL of n-heptane containing methyl myristate (30 μg/mL) was added and mixed. The GC/MS metabolomics analyses were performed as previously described. Metabolites were identified by matching against these databases: Wiley 9, the National Institute of Standards and Technology (NIST) library 14 and an in-house mass spectra library database.

#### Data analysis

The relative quantitative peak areas of each detected peak were normalized by IS before a multivariate statistical analysis using SIMCA-P software (Umetrics, Umea, Sweden). To visualize sample clustering, a partial least squares projection to latent structures and discriminant analysis (PLS-DA) were employed to process the acquired GC/MS data. The impact of ouabain on metabolic pathways was evaluated based on an online tool (http://www.metaboanalyst.ca/MetaboAnalyst/faces/Home.jsp).

### Quantitative real-time PCR analysis

Total RNA was extracted using TRIzol (Invitrogen, Thermo Fisher Scientific, MA, USA) and reverse transcribed into Complementary DNA (cDNA). Quantitative real-time polymerase chain reaction (qRT-PCR) analysis was then processed using ChamQTM SYBR qPCR Master Mix (Vazyme biotech co., Itd, China) on an ABI7500 Real-Time PCR instrument (Applied Biosystems, Inc., Thermo Fisher Scientific, CA, USA). In all experiments, the manufacturers' instructions were followed. The primer pair sequences used are listed as follows: SLC7A11, forward: 5'-TCTCCAAAGGAGGTTACCTGC-3'; reverse: 5'-AGACTCCCCTCAGTAAAGTGAC-3'; GAPDH, forward: 5'-AACAGCCTCAAGATCATCAGCA-3'; reverse: 5'-ATGAGTCCTTCCACGATACCA-3'. GAPDH was used to normalize SLC7A11 gene expression using the comparative threshold (CT) method (ΔΔCT).

### Western blot analysis

HaCaT cells were lysed in cell lysis buffer (Cell Signaling, Danvers, USA) and total proteins were extracted. Equal amounts of protein were electrophoresed on 12% polyacrylamide gels and then transferred to a 0.44-µm polyvinylidene fluoride membrane (Invitrogen). After blocking with 5% BSA, membranes were probed with antibody against SLC7A11 (26864-1-AP, Proteintech, Wu Han, China) or beclin-1 (D40C5, 3495, Cell Signaling) at 1:1000 dilutions. GAPDH was used as internal control (2118, Cell Signaling). The membranes were then incubated with secondary antibody conjugated to horseradish peroxidase (Cell Signaling) at 1:1000 dilutions, and the blots were visualized with enhanced chemiluminescence. Scanned densitometry and protein density calculation was performed using ImageJ.

### Determination of intracellular glutathione levels

The changes in the levels of reduced (GSH) and oxidized (GSSG) glutathione were determined using GSSG/GSH Quantification Kit II (G263, Dojindo, Japan) following their manuscript. Briefly, cells were collected and washed with PBS. Then 80 μL of 10 mM HCl was used to lyses cells and centrifuged (8000 *g*, 10 min). Supernatants were divided into two groups to detect GSSG and total glutathione separately. GSH levels were calculated using the following formula: GSH = total glutathione − GSSG × 2.

### Detection of cellular reactive oxygen species

The cells were resuspended in a six-well plate. After the cells adhered, they were treated with 200 nM ouabain. After 24 h, the medium was discarded, and the cells were washed with PBS twice. The fluorescent probe DCFH-DA (KGT010-1, KeyGENBioTECH, Nanjing, China) was diluted 1:1000 with DMEM, and 1 mL of the mixture was added to each well. The cells were then incubated for 20 min at 37 °C, before washed with PBS twice. After digesting with trypsin, the cells were collected and resuspended in 200 μL of PBS. Flow cytometry was used for detection of ROS.

### Determination of Akt and mTOR phosphorylation levels

The levels of phosphorylated and total Akt and mTOR were determined by a colorimetric method using the Phospho-Akt/GSK3β/mTOR ELISA kit (ab279732, Abcam) according to the manufacturer’s instructions. Akt and mTOR phosphorylation was detected by rabbit anti-phospho-AKT (S473) antibody and rabbit anti-phospho-mTOR (S2448) antibody.

### Cystine uptake assay

The extent of cystine uptake was determined using the Cystine uptake assay kit (UP05, Dojindo laboratories) following the manufacturer’s instructions. Fluorescence signal was read by MDC FlexStation II (Molecular Devices, Sunnyvale, USA; Ex = 485 nm, Em = 535 nm). Results were reported as ratio relative to control.

### Statistical analysis

All experiments were performed in triplicate and were repeated at least three times (n ≥ 3). Results are presented as mean ± SD. Statistical significance was determined using the unpaired, two-tailed, Student’s *t*-test using the Sigma-Plot 9.0 (SPSS, Chicago, USA). Significant differences were considered based on *p*-value < 0.05.

## Results

### Ouabain potently decreased keratinocyte growth

**Fig. 1:**
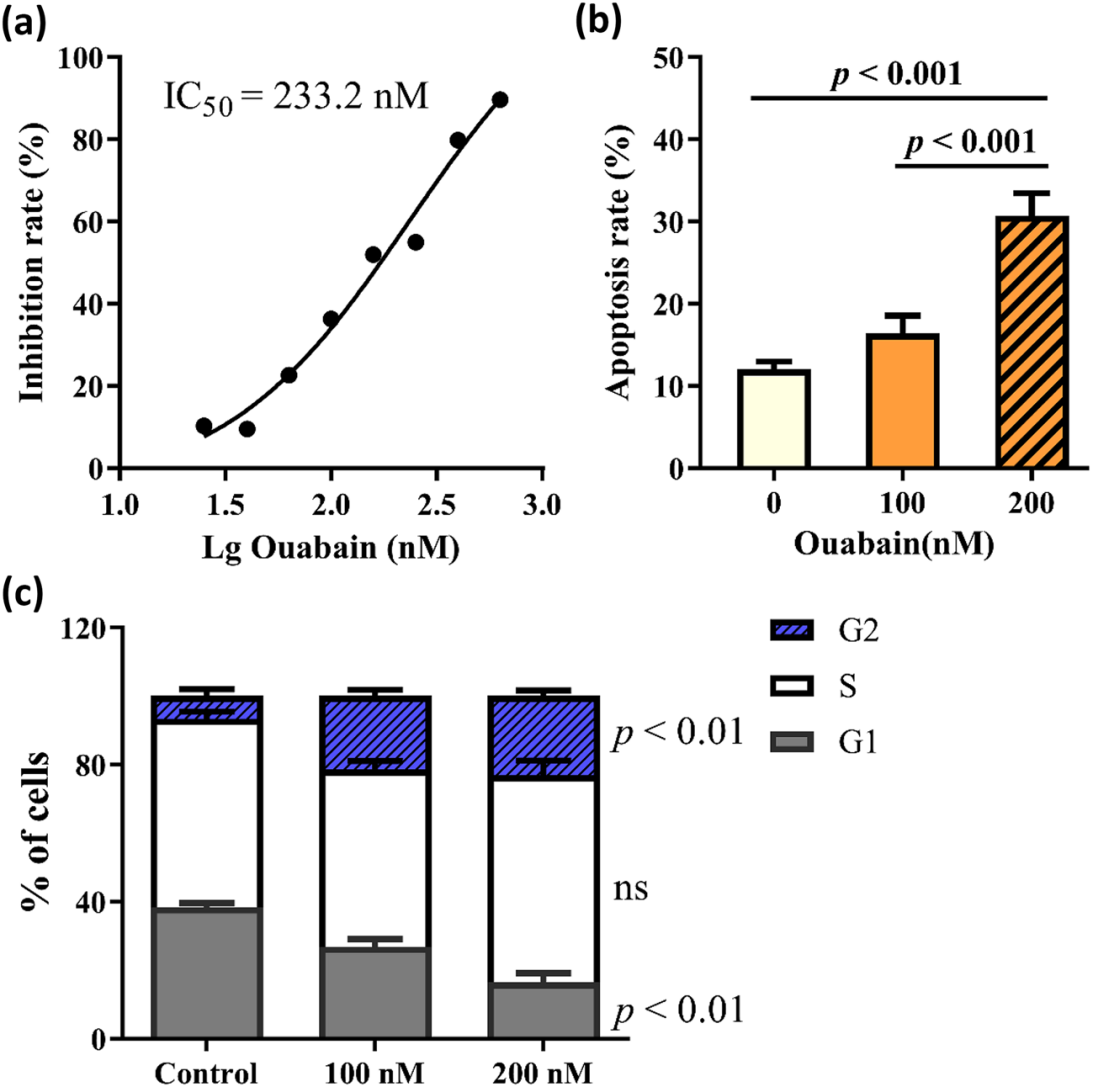
Ouabain potently decreased keratinocyte growth.** a** The proliferation of cells was assessed by the CCK-8 assay. **b** Apoptosis was determined by flow cytometry. **c** The percentage of cells in each cell cycle is represented by a bar graph. Data were presented as mean ± SD. *P*-values were calculated using the unpaired Student’s *t*-test (ns, not significant)

Ouabain significantly decreased HaCaT cell viability at nanomolar concentrations with an IC_50_ value of 233 nM (Fig. 1a). In addition, ouabain treatment (200 nM, 24 h) dramatically increased the percentage of apoptotic cells by 2.5-fold compared to control cells (Fig. 1b). At the same time, we also examined effects on cell cycle progression. As shown in Fig. 1c, ouabain treatment (200 nM, 24 h) induced G2/M cell cycle arrest. The percentage of cells that accumulated in G2/M phase increased to 23.2% after ouabain treatment as compared to only 6.7% of control cells.

### Ouabain significantly perturbed cellular metabolism

**Fig. 2:**
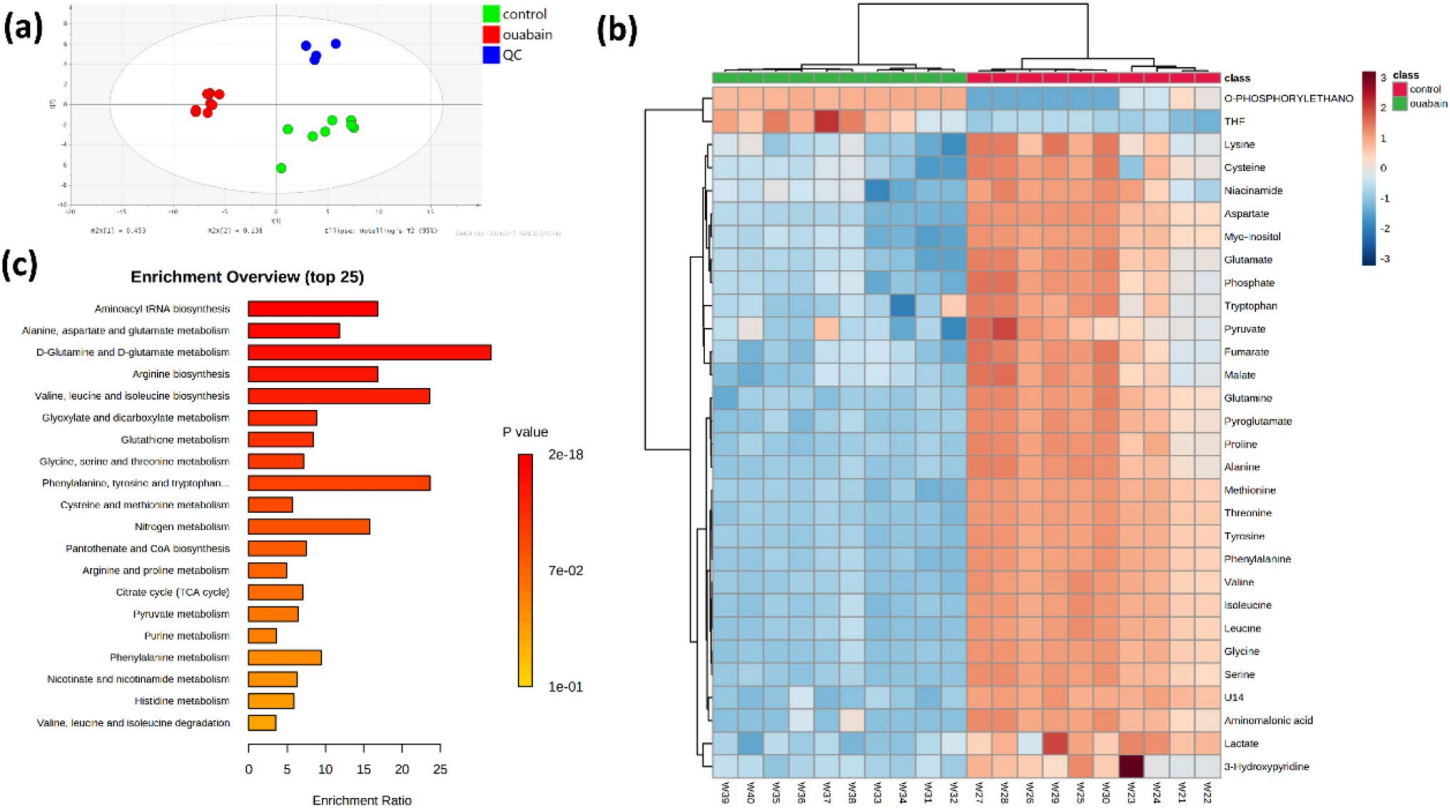
Ouabain significantly perturbed metabolomics profile of cell pellets.** a** PLS-DA score plots showing individual samples from control (green dots) and ouabain-treated (red dots) cells. The blue dots are quality control samples. **b** Heat map of metabolites with different abundance between control and ouabain-treated cells. **c** Metabolic pathway enrichment analysis based on differential intracellular metabolites

We first prepared samples from control and ouabain-treated cell pellets. The GC–MS analysis identified multiple cellular metabolites, including amino acids, small organic acids, carbohydrates, lipids and amines. After applying the partial least squares orthogonal projection to latent structure discriminant analysis (PLS-DA, Fig. 2a, b), the control cells and ouabain-treated cells clustered closely within each group and separately from each other, indicating that the metabolic patterns were significantly perturbed when HaCaT cells were exposed to ouabain. Enrichment analysis demonstrated that ouabain treatment significantly affected the following metabolic pathways (Fig. 2c): amino acid metabolism or protein biosynthesis (valine, leucine, isoleucine, proline, serine, threonine, aspartate, phenylalanine, tryptophan, lysine, tyrosine, glutamine, aminomalonic acid), glutathione metabolism (cysteine, glutamate, glycine, methionine), and carbon metabolism pathways (glyoxylate and dicarboxylate metabolism, citrate cycle) to a lesser extent (Fig. 2b).

**Fig. 3:**
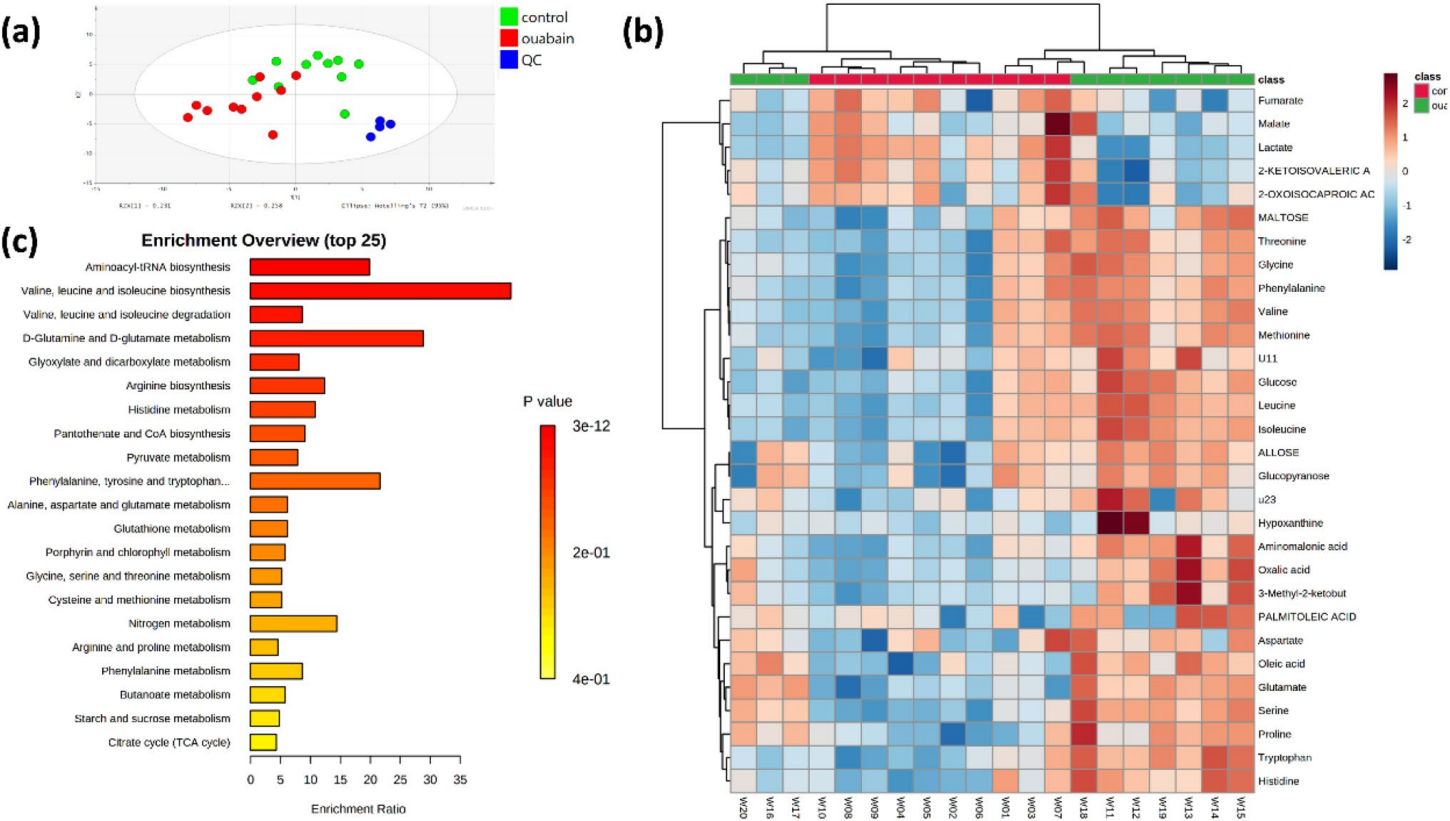
Ouabain altered metabolomics profile of cell culture supernatants. **a** A PLS-DA plot of individual samples of control (green dots) and ouabain-treated (red dots) cells. Samples of quality control are shown in blue. **b** A heatmap comparing metabolites with different abundance levels in control and ouabain-treated cells. **c** Metabolic pathway enrichment analysis based on differential culture media supernatant metabolites

Metabolic pattern of the cell culture supernatant also changed markedly (Fig. 3a, b). Major metabolic pathways affected were amino acid metabolism, glutathione metabolism, and glyoxylate and dicarboxylate metabolism (Fig. 3c). Consistent with the intracellular results, ouabain significantly increased the abundance of amino acids in the culture supernatant, including valine, leucine, isoleucine, proline, serine, threonine, aspartate, phenylalanine, tryptophan, glutamate, glycine, methionine, and aminomalonic acid, indicating less consumption of these nutrients in ouabain-treated cells (Fig. 3b). However, relative to the intracellular metabolites, the discriminatory metabolite number in the culture supernatant and their fold changes were less perturbed by ouabain treatment, probably due to the dilution factor of culture media.

### Ouabain markedly reduced glutathione levels

**Fig. 4:**
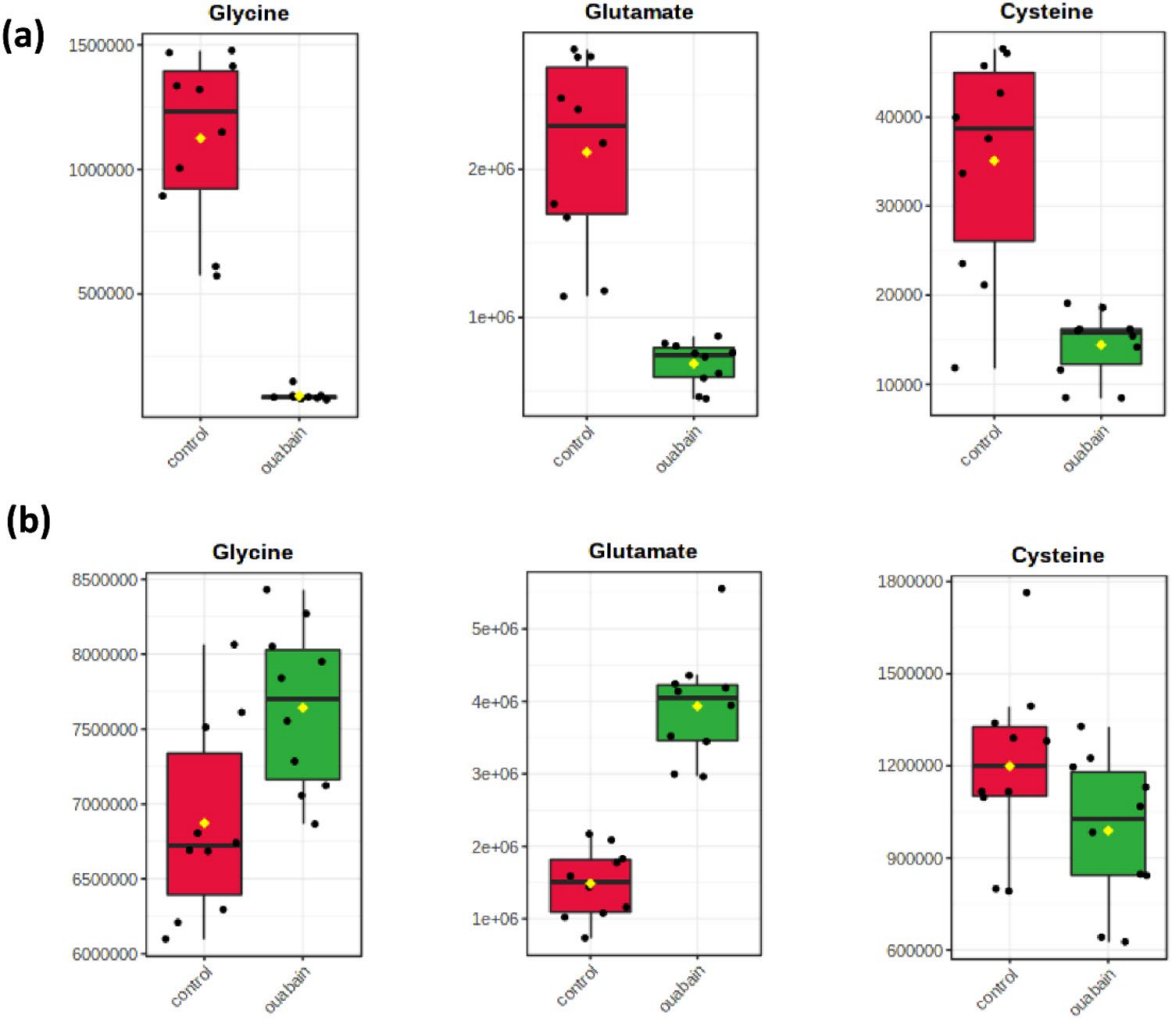
Relative abundance of typical metabolites related to glutathione synthesis. **a** Cell pellet metabolites. **b** Cell culture supernatant metabolites. Data were presented as mean ± SD. *p < 0.05, **p < 0.01, ***p < 0.001, compared to control cells

**Fig. 5:**
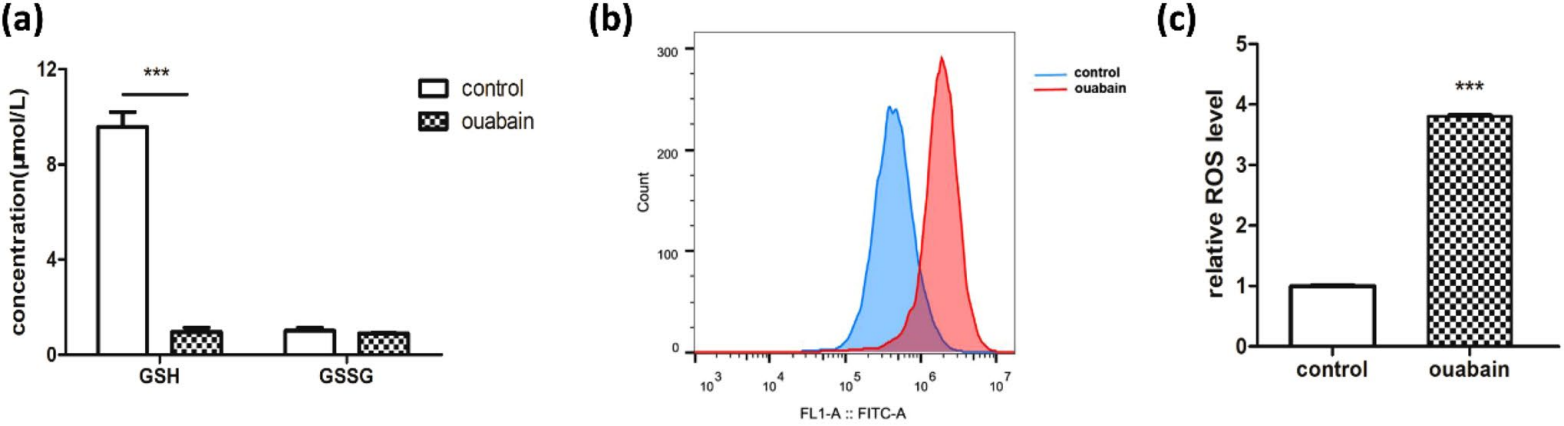
Ouabain reduced GSH and increased intracellular ROS levels. **a** Effect of ouabain on GSH and GSSG levels in HaCaT cells. **b** Cellular ROS was assessed with flow cytometry. **c** A bar plot reflects the increase of ROS levels. ***p < 0.001

Considering the important role of oxidative stress and glutathione (GSH) metabolism in psoriasis (Yildirim et al. 2003; Dobrică et al. 2022), we focused on the alteration of this metabolic pathway. Metabolism analysis showed that the abundance of three precursors of GSH (cysteine, glutamate, and glycine) was markedly reduced in ouabain-treated cells (Fig. 4a). On the contrary, the abundance of glycine and glutamate were elevated in the culture media, except for cysteine which was still lower than the control group (Fig. 4b). As seen in Fig. 5a, the concentration of GSH decreased significantly by almost 90% with no obvious change of oxidized glutathione (GSSG). The ratio of GSH/GSSG was also decreased, indicating reduced consumption of cysteine and reduced GSH synthesis. This could also be due to enhanced utilization of the reduced form of GSH to combat the onset of oxidative stress.

### Ouabain increased intracellular ROS levels

The main effect of the anti-oxidant GSH is to maintain ROS levels within cells. We next examined if ouabain could interfere with intracellular ROS levels. Results of flow cytometry showed that ROS levels were up-regulated significantly by threefold after ouabain treatment (Fig. 5b and c), indicating that ouabain induced oxidative stress, probably through inhibition of GSH synthesis.

### Ouabain decreased protein levels of cysteine transporter SLC7A11

**Fig. 6:**
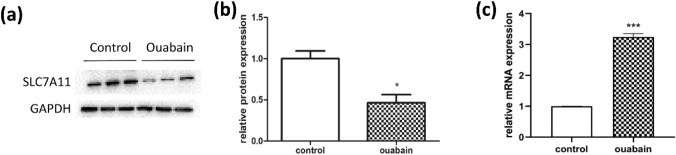
Ouabain decreased the protein level of cysteine transporter SLC7A11. **a, b** SLC7A11 protein expression was determined by Western blots and results were shown in bar plots. **c** The mRNA expression of SLC7A11 was measured by q-RT PCR after ouabain treatment. *p < 0.05, ***p < 0.001

Cysteine is a rate-limiting precursor for GSH synthesis. Since ouabain reduced GSH levels and the abundance of intracellular cysteine, we wondered if ouabain could interfere with cysteine uptake. Indeed, we found a significant decrease in the protein level of cysteine transporter SLC7A11 by about 50% after ouabain treatment, despite an elevation in its mRNA expression (Fig. 6). We speculate that ouabain might interfere with post-translational regulation of SLC7A11, and its mRNA expression was increased for compensation.

### Ouabain downregulated SLC7A11 protein via Akt/mTOR/beclin axis

**Fig. 7:**
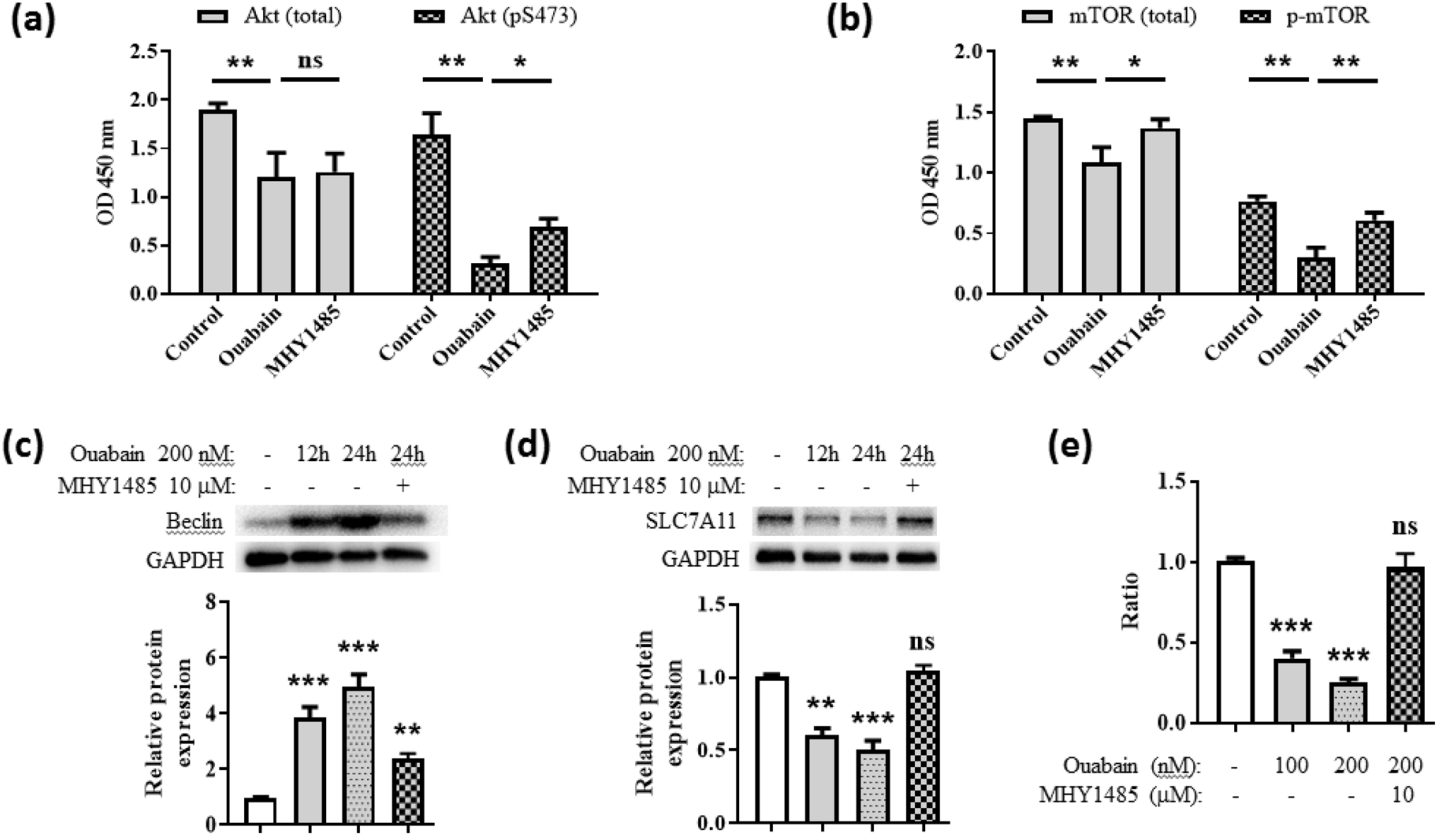
Ouabain downregulated SLC7A11 protein via Akt/mTOR/beclin axis. The total and phosphorylated levels of **a** Akt and **b** mTOR were determined by a colorimetric method using the Phospho-Akt/mTOR ELISA kit. Beclin (**c**) and SLC7A11 (**d**) protein levels were determined by Western blots. Cells were treated with 200 nM ouabain for 12–24 h, with or without MHY1485 pretreatment (10 μM, 30 min). **e** Cystine uptake was measured using a commercialized kit. *p < 0.05, **p < 0.01, ***p < 0.001, ns, not significant

A previous study has shown that the mammalian target of rapamycin (mTOR) pathway regulates SLC7A11 activity and inhibition with Torin 1 reduced the protein levels of SLC7A11 (Yamaguchi et al. 2020). Other studies have reported that beclin can block SLC7A11 activity by directly binding SLC7A11 to form a complex (Kang et al. 2018; Song et al. 2018). Since mTOR is a known negative regulator of beclin (Nikoletopoulou et al. 2013), we wondered if ouabain changes SLC7A11 protein expression via mTOR-beclin axis. We measured the phosphorylation levels of Akt and mTOR after ouabain treatment in keratinocytes. Our results showed that phospho-Akt (S473) (Fig. 7a) and phospho-mTOR (S2448) (Fig. 7b) levels were significantly decreased by ouabain, which rebounded after pretreatment with the mTOR activator MHY1485. Beclin levels significantly increased after ouabain treatment and was more prominent at 24 h than 12 h, but beclin was inhibited by MHY1485 pretreatment (Fig. 7c). Accordingly, SLC7A11 protein declined along with the increase of beclin, whilst SLC7A11 protein levels recovered when beclin was inhibited (Fig. 7d). Lastly, we verified cysteine uptake as an indicator of SLC7A11 protein activity. Ouabain inhibited cysteine uptake by more than 50%, but MHY1485 pretreatment diminished this effect (Fig. 7e).

## Discussion

We explored the effects and mechanism of ouabain on cell growth and metabolism in human keratinocytes using untargeted GC/MS metabolomics approach. Ouabain significantly inhibited human psoriatic keratinocyte growth, which has never been reported before. Moreover, ouabain dramatically impaired glutathione metabolism, also affected amino acid metabolism and some carbon metabolism pathways. Mechanistically, ouabain downregulated cystine transporter SLC7A11, reduced glutathione synthesis and caused oxidative stress, probably by inhibiting the Akt/mTOR/beclin signaling pathway that is related to the protein activity of SLC7A11. The altered cellular metabolism and oxidative stress caused by ouabain may contribute to its cytotoxic effects toward keratinocytes.

Ouabain rapidly decreased proliferation, induced apoptosis, and caused G2/M cell cycle arrest of keratinocytes. Our observations are supported by recent evidence demonstrating cytotoxic effects of ouabain in cancer cells that are also rapidly-proliferating. Ouabain inhibits proliferation of Burkitt’s lymphoma Raji cells (Meng et al. 2016), and causes cell cycle arrest at G2/M stage in myeloid leukemia cells without obvious cytotoxicity on normal blood cells (Feng et al. 2016). Excessive proliferation and reduced apoptosis of keratinocytes are the main characteristics of psoriasis (Zampetti et al. 2009). Some common psoriasis treatments, such as methotrexate and dithranol, exert their therapeutic effects by counteracting keratinocyte hyperproliferation and inducing apoptosis (Griffiths et al. 2021). According to the anti-proliferative and pro-apoptotic effects of ouabain on keratinocytes, it could be potentially useful for treating hyperproliferative skin diseases, such as psoriasis.

It’s worth noting that ouabain concentrations used in this study are below the well-documented concentration needed to induce its housekeeping ion pump function (Aizman et al. 2001). It has been demonstrated that low doses of ouabain (10 nM–100 μM) can stimulate ROS generation and NFkB activation, which is not related to inhibition of Na-K-ATPase activity nor intracellular Na + or K + concentrations (Liu et al. 2000; Aydemir-Koksoy et al. 2001; Aizman and Aperia 2003). Moreover, ouabain can modulate epithelial cell tight junctions through ERK1/2 and c-Src signaling pathways (Rajasekaran et al. 2001; Larre et al. 2010), suggesting that ouabain has signaling functions independent of Na-K-ATPase inhibition.

For psoriatic keratinocytes, it is necessary to reprogram cellular metabolism to meet the needs of rapid proliferation (Cibrian et al. 2020). We thus examined the effects of ouabain on cellular metabolism. Ouabain markedly inhibited glutathione metabolism and amino acid metabolism, also reduced carbon metabolism pathways (i.e. glyoxylate and dicarboxylate metabolism, citrate cycle) to a lesser extent. This indicated that ouabain may have successfully entered the cells and dramatically impaired the above-mentioned metabolic pathways. Previous studies have reported increased serum levels of amino acids in psoriatic patients that also positively correlate with clinical PASI score (Kamleh et al. 2015; Kang et al. 2017). Amino acids are the building block of proteins and participate in various cellular processes, such as energy production. Here we showed that ouabain significantly decreased the absorption of various amino acids in keratinocytes and impaired cell metabolism from the source.

More importantly, ouabain significantly inhibited glutathione metabolism, reduced intracellular levels of cysteine and glutathione, and induced oxidative stress. Glutathione is synthesized from cysteine, glutamate and glycine. Glutathione is a key player in maintaining antioxidant defense systems inside the cell (Lin et al. 2020). It has been shown that glutathione synthesis is significantly upregulated in psoriasis in order to reduce the generation of ROS accompanying energy production (Campione et al. 2022); and methotrexate treatment can inhibit glutathione synthesis, induce oxidative stress and promote apoptosis of keratinocytes (Zong et al. 2020). This is in consistent with our results and reconfirms that glutathione metabolism is a critical player during psoriasis pathogenesis.

Considering the importance of glutathione synthesis and oxidative stress in psoriasis, we next focused on this pathway. So how does ouabain impact glutathione metabolism? The rate of glutathione synthesis is primarily limited by intracellular cysteine content (Zhu et al. 2019). Cysteine is transported to the intracellular space through a heterodimeric cysteine/glutamate antiporter system, which contains a critical catalytic subunit solute carrier family 7 member 11 (SLC7A11) (Lin et al. 2020). Pharmacological inhibition of the transporter system leads to ROS accumulation and cell death (Zhu et al. 2019). More importantly, SLC7A11 is positively correlated with psoriasis (Cibrian et al. 2020). According to our results, ouabain potently reduced protein levels of SLC7A11 despite an increase in its mRNA expressions, suggesting post-translational downregulation of SLC7A11 by ouabain. There might be a compensatory increase of SLC7A11 mRNA expression in order to counter balance the elevated levels of oxidative stress. Indeed, studies have reported that intracellular accumulation of ROS upregulates SLC7A11 mRNA expression as a protective response (Ishimoto et al. 2011; Ali et al. 2016).

The next question is: how dose ouabain downregulate SLC7A11? Evidence regarding the regulatory mechanisms of SLC7A11 protein activity and stability is scarce. It has been reported that mTOR activation increases SLC7A11 protein levels by suppressing lysosomal degradation in glioblastoma cells, and inhibition with Torin 1 reduced the protein levels of SLC7A11 (Yamaguchi et al. 2020). In addition, Cheng et al*.* have demonstrated that ouabain suppresses the Akt/mTOR signaling pathway and inhibits the growth and motility of U-87MG human glioma cells (Yang et al. 2018). Other studies have reported that beclin can block SLC7A11 activity by directly binding SLC7A11 to form a complex, and is independent of autophagy (Kang et al. 2018; Song et al. 2018). Since mTOR is a known negative regulator of beclin (Nikoletopoulou et al. 2013), we examined if ouabain changed SLC7A11 protein expression via Akt/mTOR/beclin axis. As expected, ouabain significantly inhibited Akt and mTOR phosphorylation, increased beclin, reduced SLC7A11 protein expression and blocked cystine uptake. Pretreatment with the mTOR activator MHY1485 significantly reversed these effects, suggesting that ouabain may downregulate SLC7A11 protein and cystine uptake via Akt/mTOR/beclin axis. This is supported by previous evidence that metabolic pathways can regulate keratinocyte activation through mTOR (Cibrian et al. 2020).

**Fig. 8:**
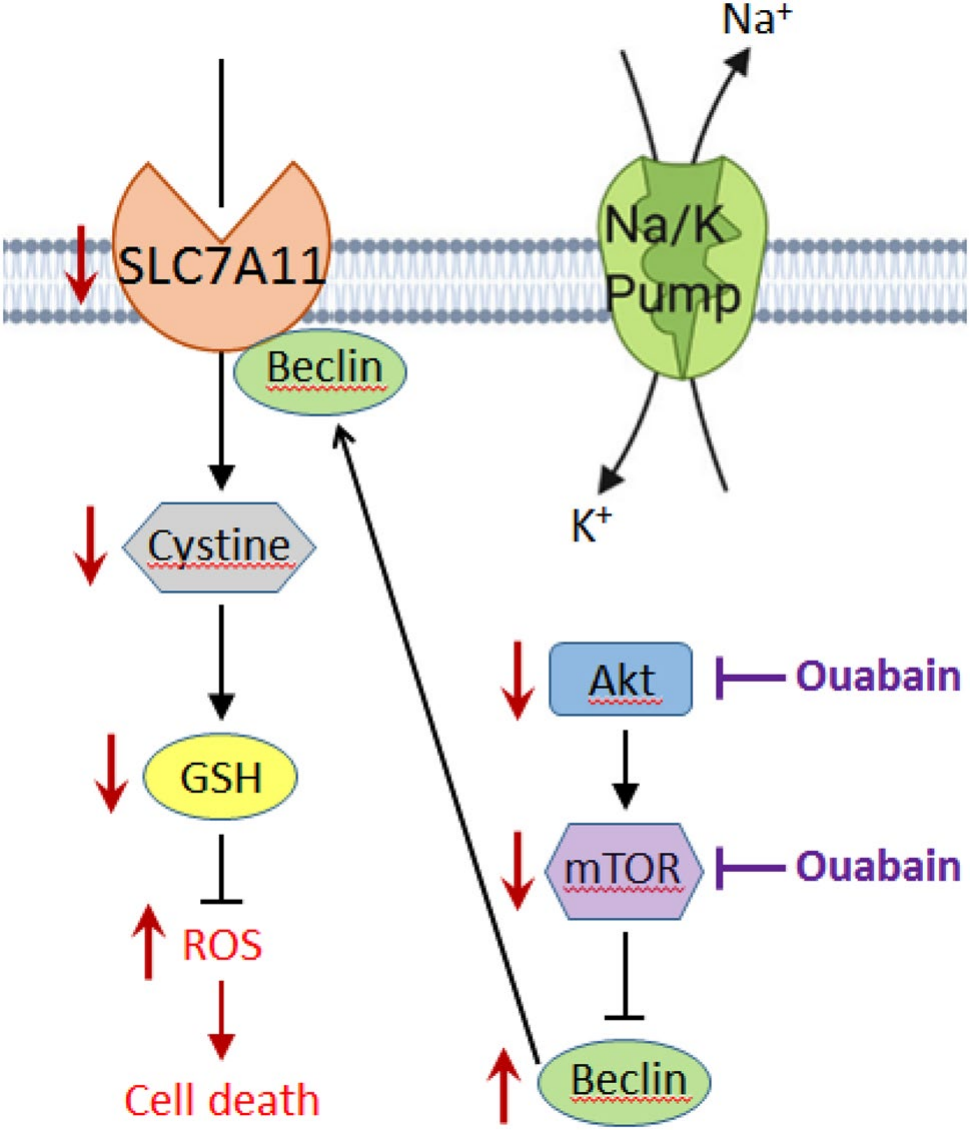
A summary figure showing mechanism of ouabain cytotoxicity towards keratinocytes. Low doses of ouabain cause no detectable inhibition of the Na + /K + pump activity, but inhibits activation of Akt and mTOR, which leads to upregulation of beclin. Beclin forms a complex with SLC7A11 and directly reducing its protein expression, decreasing cystine uptake. Consequently, intracellular GSH synthesis declines, causing excessive oxidative stress and ultimate cell death

In conclusion, we report for the first time that ouabain caused growth arrest of human psoriatic keratinocytes mainly by impairing GSH synthesis and redox status. As depicted in Fig. 8, ouabain inhibits Akt and mTOR phosphorylation, which then upregulates beclin levels. Beclin directly forms a complex with SLC7A11 and decreases its protein activity. As a result, cystine uptake drops and intracellular GSH synthesis declines, causing excessive oxidative stress and ultimately cell death. Our study not only provides experimental evidence supporting the role of SLC7A11-mediated cystine uptake and GSH metabolism in keratinocyte proliferation, but also suggests ouabain as a potential anti-psoriatic agent by inhibiting the Akt/mTOR/beclin axis. Further metabolomic studies of psoriasis patients are required to verify changes of specific metabolites and metabolic pathways that participate in psoriasis pathogenesis.
